# Comparative Evaluation of Apical Leakage in Root Canal Obturation Using AH Plus Sealer, Bioceramic Sealer, and Bioceramic Sealer Incorporated With Chitosan Nanoparticles: An In Vitro Study

**DOI:** 10.7759/cureus.75359

**Published:** 2024-12-09

**Authors:** Sushmita Rane, Varsha Pandit, Sanpreet S Sachdev, Shivani Chauhan, Rishabh Mistry, Barun Kumar

**Affiliations:** 1 Conservative Dentistry and Endodontics, Bharati Vidyapeeth (Deemed to be University) Dental College and Hospital, Pune, IND; 2 Oral Pathology and Microbiology, Bharati Vidyapeeth (Deemed to be University) Dental College and Hospital, Pune, IND; 3 Dentistry, D.Y. Patil (Deemed to be University) School of Dentistry, Navi Mumbai, IND; 4 Oral and Maxillofacial Surgery, Bharati Vidyapeeth (Deemed to be University) Dental College and Hospital, Pune, IND

**Keywords:** apical leakage, bioceramic sealer, chitosan nanoparticles, endodontic re-infections, microleakage, obturation, resin sealer, resin sealer, root canal sealers

## Abstract

Introduction

Endodontic re-infections primarily occur due to the ingress of bacteria and their toxins through an incomplete seal following obturation. A variety of sealers have been developed to achieve effective integration with the different obturation materials and dentinal tubules. To choose the right endodontic sealer and application for each clinical instance, one must be aware of the attributes of the various sealers commonly used in clinical practice. The utility of chitosan nanoparticles in endodontics requires exploration to understand the faring of this material in comparison to the existing gold standards. This study aims to compare the apical leakage after obturation using AH Plus sealer and bioceramic sealer with and without chitosan nanoparticles.

Materials and methods

Forty single-rooted, single-canal extracted teeth were selected and decoronated to standardize the root length to 14 mm. Root canals were prepared using Protaper rotary files and irrigated with sodium hypochlorite, EDTA (ethylenediaminetetraacetic acid), and saline. The samples were randomly divided into four groups (n=10): group I (gutta percha), group II (gutta percha + AH Plus sealer), group III (gutta percha + bioceramic sealer), and group IV (gutta percha + chitosan nanoparticles in bioceramic sealer). The sealers were applied, and obturation was performed using the cold lateral condensation technique. Post-obturation, the samples were sealed with Cavit-G, coated with nail varnish, and immersed in 2% methylene blue dye for 72 hours. The teeth were then sectioned longitudinally and inspected for dye penetration using a stereomicroscope.

Results

The mean dye penetration values were as follows: 4.77 ± 2.08 mm in group I, 2.93 ± 0.40 mm in group II, 2.34 ± 1.46 mm in group III, and 1.93 ± 1.68 mm in group IV. Statistical analysis using one-way ANOVA showed significant differences (p < 0.05) between the groups. Tukey’s post hoc test revealed the least microleakage in group IV, indicating superior sealing ability of bioceramic sealer with chitosan nanoparticles.

Conclusion

Incorporation of chitosan nanoparticles in bioceramic sealer significantly enhances its sealing ability, reducing apical microleakage more effectively than gutta percha (2.5 times), AH Plus sealer (1.5 times), and bioceramic sealer alone (1.2 times). This suggests that chitosan nanoparticles could be a promising addition to endodontic sealers to improve treatment outcomes.

## Introduction

Endodontic re-infections occur due to thriving of the bacteria and their toxins in the root canal following endodontic treatment. The most frequent cause for re-infection is the lack of a complete seal following obturation enabling the ingress of bacteria into the canal [1]. These events consequently lead to endodontic failure, resulting in a decline in the patient’s quality of life as well as confidence in the dentist. It is, thus, imperative to prevent the occurrence of re-infections, for which numerous measures are adopted by endodontists. Numerous obturation materials and techniques have been devised over the past years to minimize this re-infection due to microleakage. Even so, the phenomenon of root canal failure due to apical leakage is a fairly common occurrence in endodontic practice [2,3].

In routine practice, sealers are adapted to the walls of the canal that block the dentinal tubules by flowing into them and also lubricate the core filling material, usually gutta percha. For a fluid-tight closure, the ideal root canal sealer should have enhanced wettability, low surface tension, and biocompatibility for efficient penetration into defects. A variety of sealers have been developed to achieve effective integration with different obturation materials and dentinal tubules. However, to achieve a three-dimensional seal, the sealer has to combine chemically with the core material as well as the dentinal walls [4,5]. Microleakage in root canal sealers depends on various factors such as sealer composition, sealer penetration and adaptation, sealer flowability, sealer setting time, obturation technique, moisture contamination, and root canal anatomy [6-8]. A recent study demonstrated that inadequate sealer function accounts for around 60% of endodontic failures [6].

To choose the right endodontic sealer and application for each clinical instance, one must be aware of the attributes of the various sealers commonly used in clinical practice. Various commonly employed sealers include resin, mineral trioxide aggregate, bioceramic, zinc oxide eugenol, and calcium hydroxide [9]. It has been shown that epoxy resin sealers are associated with less apical microleakage than eugenol-based and zinc oxide sealers. The AH Plus (Dentsply Sirona, Charlotte, NC, USA), a resin-based sealer, has been recognized as a gold standard, given its adaptation to the root canal walls, and physical/chemical and biological qualities [10]. Bioceramic, a biocompatible material containing calcium phosphate and calcium silicate, has gained popularity as a sealer among endodontists in recent times. It has a crystalline structure and chemical characteristics comparable to those of tooth and bone apatite materials. The material has vastly improved sealing capacity, which almost guarantees a successful treatment outcome [11].

A relatively novel material used in endodontic sealers is chitosan, a cellulose-like biopolymer and a chemically altered version of chitin found in the exoskeleton of several insects and marine animals [12]. The material has an inherent antibacterial action, which is augmented by formulating nanoparticles. When compared to their bulk counterparts, nanoparticles of chitosan have unique physicochemical traits including huge surface area/mass ratios, greater chemical reactivity, and ultra-small sizes [13]. Because of their hydrophilic nature, these particles provide a bioadhesive that adsorbs better on the dentinal wall, improving the sealer's capacity to seal [13,14].

The utility of chitosan nanoparticles in endodontics requires exploration to understand the faring of this material in comparison to the existing gold standards. Hence, the present study was conducted to compare the apical leakage after obturation using the AH Plus sealer and bioceramic sealer with and without chitosan nanoparticles.

## Materials and methods

Single-rooted teeth extracted for orthodontic/periodontal reasons were considered eligible for inclusion in the present study. Only non-carious teeth with a single root canal and fully developed roots were included. The soft tissues, dental calculus, and stains were removed from the teeth and stored in normal saline till further use.

Sample preparation

**Figure 1:**
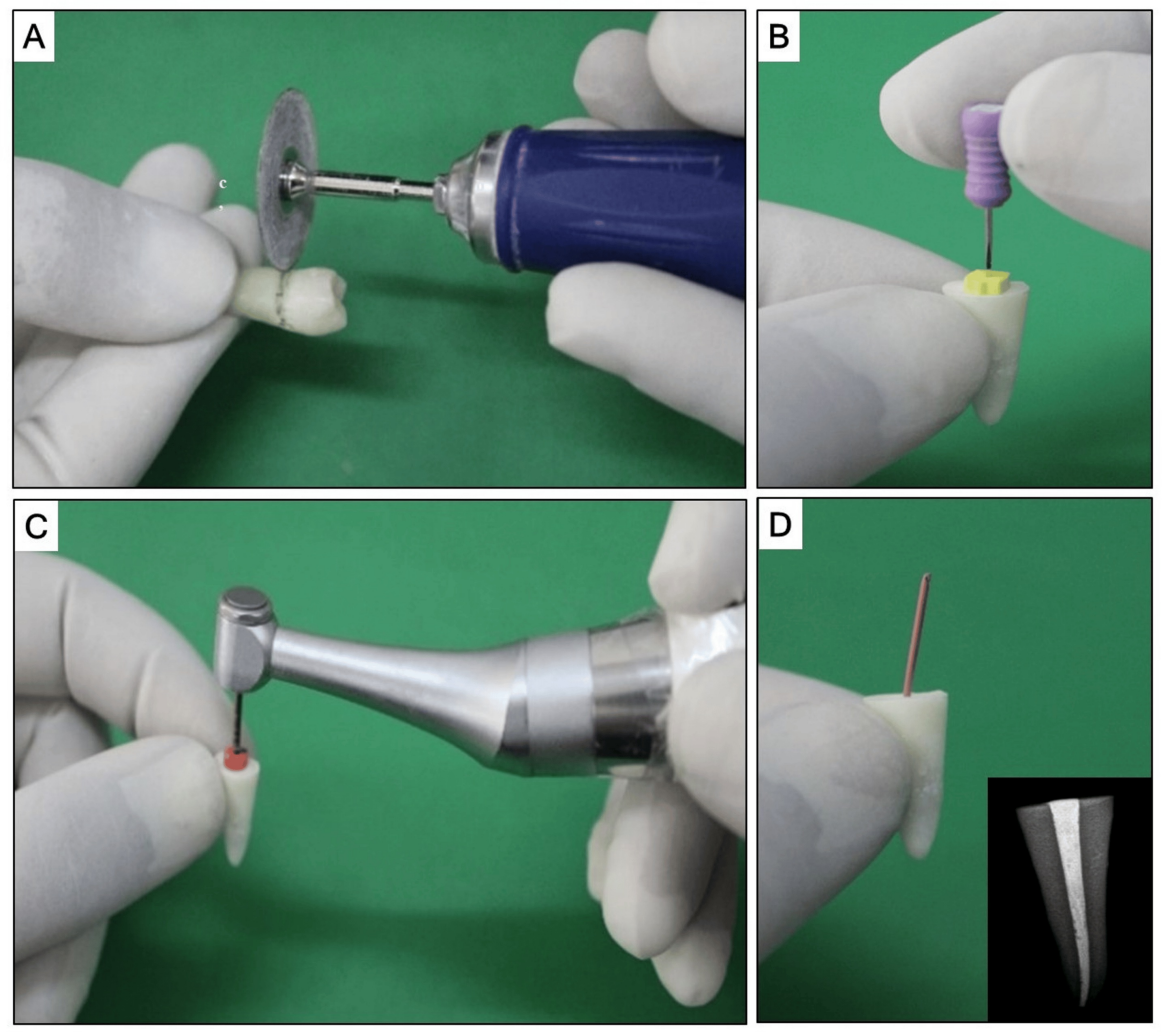
(A) Decoronation of the tooth at the CEJ level. (B) Determination of working length. (C) Preparation of the canals with Protaper rotary files. (D) Selection of appropriate master cone (inset: radiographic confirmation). CEJ, cementoenamel junction

All specimens were decoronated at the cementoenamel junction level with a diamond disc under water coolant. To standardize the root length of 14 mm, the teeth were decoronated at the cementoenamel junction level using a slow-speed diamond disc while continuously cooled by water. An endo-access bur was used for access opening. Patency of the canal was obtained with a #10 hand K-file (Mani, Tokyo, Japan), which was reinserted into each root canal until the file's tip was visible at the tip of the foramen The length was then measured and 1 mm was deducted to determine the working length. The root canals were prepared with Protaper rotary files (Dentsply Sirona) in a crown-down manner. The samples were placed intermittently in saline until completion of the preparation, and 17% EDTA (ethylenediaminetetraacetic acid) gel (RC Help, Prime Dental, Thane, Mumbai) was used as a lubricant during instrumentation. Protaper Gold rotary SX shaping file (Dentsply Sirona) was the first shaping file used, and it was moved apically to 2 mm short of the working length. S1 and S2 were then employed to shape the coronal two-thirds of the canal. Using Protaper Gold rotary finishing F1 and F2 files (Dentsply Sirona), according to the working length determined, the apical one-third of the canal was completed. Following the use of each instrument, the canals were irrigated with 10 mL of 5% sodium hypochlorite solution and 5 mL of EDTA 17%, and a #10 K file was used for recapitulation to maintain the foraminal patency. After instrumentation, the canals were lastly washed with sterile saline to get rid of any dentinal debris that might have remained. Paper points were then used to dry the canals, and an appropriate master cone was selected. The preparatory procedures are depicted in Figure 1.

Randomization and allocation

The manufacturer's recommendations were followed for handling and mixing the experimental root canal sealers. Based on the sealers used, 40 samples were randomly divided by using computer-generated codes into four groups (n=10 samples in each group) to ensure equal distribution of the potential confounding variables such as curved canals, lateral canals, and complex canal anatomy. The groups were as follows: group I (gutta percha), group II (gutta percha + AH Plus sealer), group III (gutta percha + bioceramic sealer), and group IV (gutta percha + chitosan nanoparticles incorporated in bioceramic sealer).

Application of chitosan nanoparticles incorporated in bioceramic sealer

The chitosan particles (Nano Research Lab, Jamshedpur, Jharkhand) used were spherical, of size 50-100 nm, and of 99% purity with certified quality (Appendix). By combining chitosan nanoparticles with bioceramic sealer powder in a ratio of 15 mg:100 mg, bioceramic-chitosan nanoparticles sealer was obtained. Using a spatula, this powder was combined with the liquid of bioceramic sealer till the particles were hydrated and the desired consistency was achieved. The cold lateral condensation technique was used to obturate the root canal for all samples. A master cone gutta percha (Dentsply Sirona) selected according to master apical file size and then the lateral compaction technique was carried out using 2% gutta percha cones (Dentsply Sirona) and #20 spreader (Mani). The sealer was coated onto the canal wall with master cone gutta percha, and lateral compaction was carried out using smaller accessory cones. The sealers were then left to set at 37°C in an incubator for 24 hours.

Dye application

Post-obturation radiography was performed. The coronal end of the canals was sealed with Cavit-G after the removal of 2-3 mm of coronal gutta percha, and two coats of nail varnish were applied covering the entire sample including the access restoration but leaving 2 mm exposed at the root's apical region. These samples were allowed to dry for 48 hours. After that, they remained immersed in 2% methylene blue dye for 72 hours. Lastly, the teeth were washed with tap water. After that, samples at a high speed were cut longitudinally while being continuously cooled with water using a cylindrical diamond disc, until one side of the samples had intact root canal filling.

Leakage analysis

**Figure 2:**
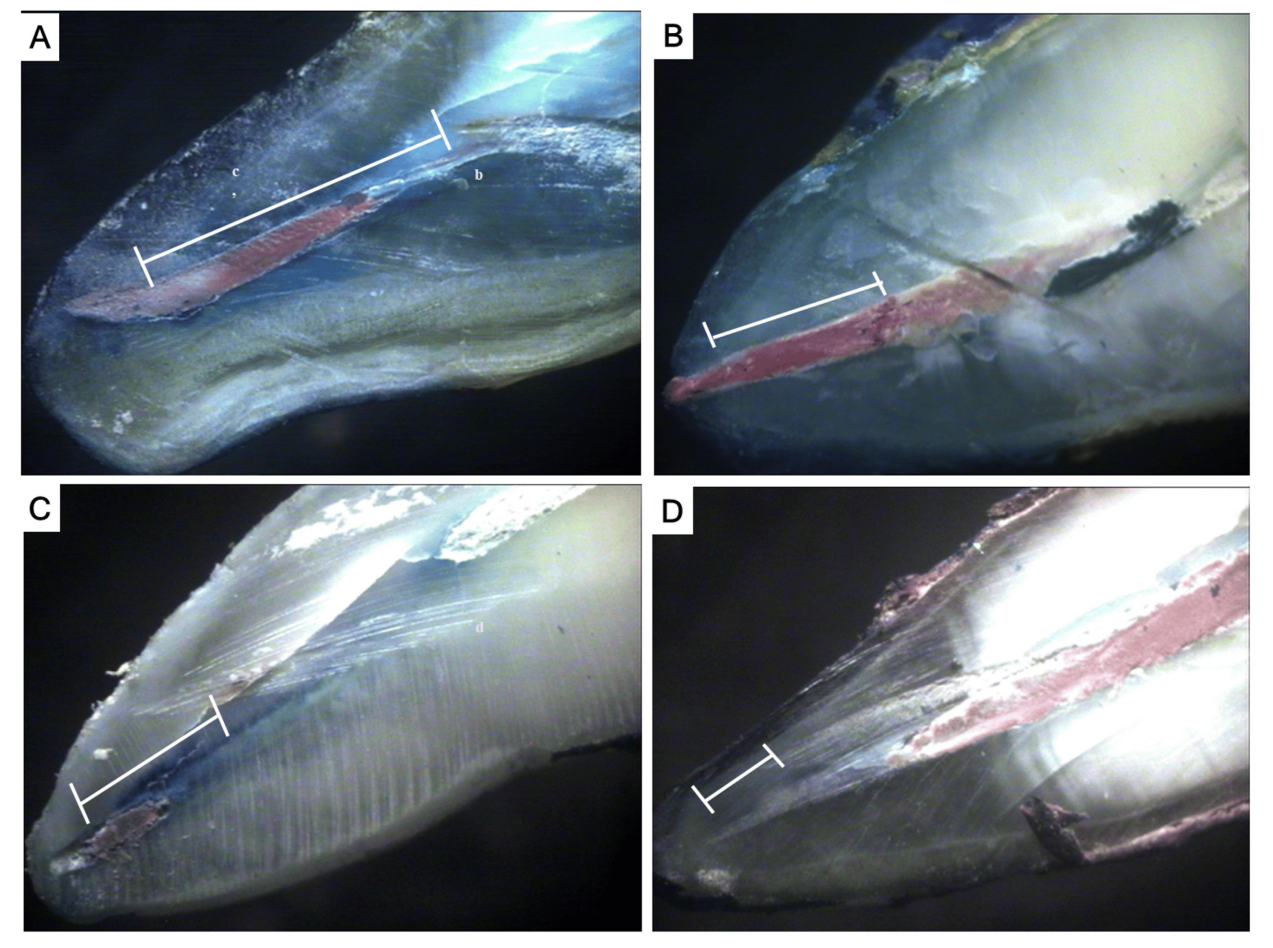
Stereomicroscopic images of the samples of (A) group I, (B) group II, (C) group III, and (D) group IV.

Sections were inspected at a 10x magnification using a stereomicroscope. The stereomicroscopic images of all the samples of each group were taken (Figure 2). using an image analysis software and the built-in scale in the stereomicroscope, the depth of dye penetration was determined (in millimeters) from the apical point to the most coronal region of the dye. The values of dye leakage measurement (measured in millimeters) were recorded, and statistical analysis was performed.

Statistical analysis

Measurement data from the dye penetration method (n=40) was analyzed using one-way analysis of variance (ANOVA), with 95% confidence level, and a p-value of less than 0.05 was accepted as statistically significant. Statistical analysis was performed using Statistical Package Social Sciences (SPSS) software (Version 21.0, IBM Corp., Armonk, NY). Tukey's post hoc test was used to conduct a post hoc multiple comparison to assess significant group differences. Results from observation were used as supporting data to observe interface morphology between root canal wall and sealer.

## Results

**Figure 3:**
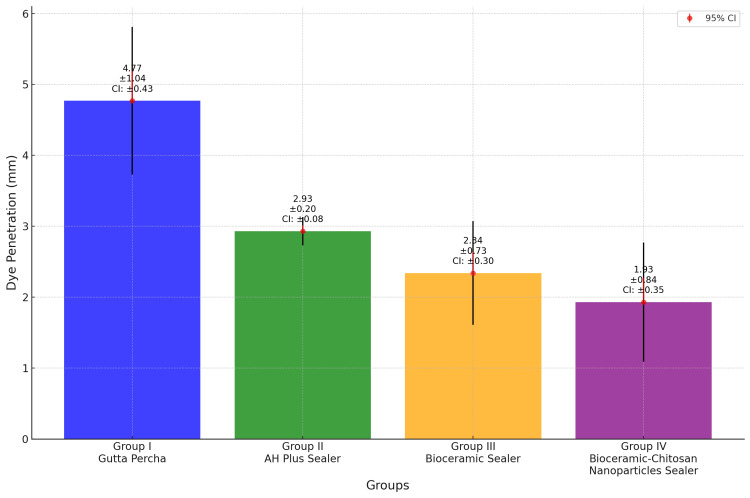
Comparative presentation of mean microleakage across the four groups

The values of dye penetration for all 10 samples of each group and their mean and standard deviation are listed in Table 1 and visually represented in Figure 3. The least mean penetration value of the dye (1.93 mm) was observed in group IV (gutta percha + chitosan).

**Table 1:** Linear methylene blue dye penetration

Sr. no.	Dye penetration (in mm)			
	Group I (gutta percha)	Group II (AH Plus sealer)	Group III (bioceramic sealer)	Group IV (bioceramic-chitosan nanoparticles sealer)
1.	6.50	3.30	2.46	0
2.	4.05	2.98	2.00	5.1
3.	6.38	2.86	0	1.32
4.	6.60	2.15	3.90	3.10
5.	0.58	3.44	4.62	0.85
6.	5.66	2.98	2.65	4.21
7.	1.80	2.67	3.56	1.92
8.	4.51	2.54	2.37	1.00
9.	6.11	2.98	1.44	0.30
10.	5.53	3.42	0.44	1.58
Mean	4.77 + 2.08	2.93 + 0.40	2.34 + 1.46	1.93 +1.68

Intergroup comparison of apical microleakage using one-way ANOVA showed a statistically significant difference (p < 0.05) between the four groups (Table 2). To better understand the differences between the respective groups, a pair-wise multiple post hoc comparison was performed using Tukey’s post hoc test. Results of the pair-wise test showed the microleakage to be highest in group I (gutta percha), followed by group II (AH Plus sealer), group III (bioceramic sealer), and group IV (chitosan nanoparticles in bioceramic sealer).

**Table 2:** Comparison of apical microleakage between different groups *Statistically significant (p < 0.05)

Comparison groups	Groups	Mean difference	p-Value
Group I (gutta percha)	Group II (AH Plus sealer)	1.8400	0.05*
	Group III (bioceramic sealer)	2.4280	0.006*
	Group IV (chitosan nanoparticles in bioceramic sealer)	2.8340	0.001*
Group II (AH Plus sealer)	Group I (gutta percha)	-1.8400	0.05*
	Group III (bioceramic sealer)	.5880	0.049*
	Group IV (chitosan nanoparticles in bioceramic sealer)	.9940	0.041*
Group III (bioceramic sealer)	Group I (gutta percha)	-2.4280	0.006*
	Group II (AH Plus sealer)	-.588	0.049*
	Group IV (chitosan nanoparticles in bioceramic sealer)	.4060	0.003*
Group IV (chitosan nanoparticles in bioceramic sealer)	Group I (gutta percha)	-2.8340	0.001*
	Group II (AH Plus sealer)	-.9940	0.041*
	Group III (bioceramic sealer)	-.4060	0.003*

## Discussion

The objective of the present study was to compare microleakage occurring after obturation of the root canals with gutta percha alone or in combination with AH Plus and bioceramic sealer with/without chitosan nanoparticles. Studies suggest that the lower density and width of dentinal tubules at the apical level may be the reasons for the difference in the leakage between the apical and coronal levels, as well as the existence of a smear layer, which results in decreased sealer penetration [15,16]. Despite improvements in sealers, complete eradication of leakage is not possible due to anatomical variations at the apical third. As a result, in this in vitro investigation, we assessed microleakage in the apical third area.

The ability of obturating materials to seal the dentinal wall is assessed through various tests, such as fluid filtration, electrochemical leakage, radioactive isotope penetration, dye penetration, and scanning electron microscopy [17,18]. Organic dyes, particularly methylene blue, are a traditional and widely used method for evaluating microleakage. This inexpensive and simple passive technique involves the movement of capillary fluid into the dentinal tubules. Grossman first used dye penetration to detect leakage in temporary filling materials, and Stewart later used methylene blue for studying microleakage [19,20]. Methylene blue dye is effective because it is quickly absorbed, non-reactive with hard tissue, and highly visible under light. The duration of dye immersion for testing ranges from one day to six months, and in the present study, the samples were immersed in methylene blue for 72 hours. The dye penetration is then visualized under high magnification using a stereomicroscope as was done in the present study [21].

Bioceramic sealer with or without the incorporated particles was found to be superior to AH Plus in terms of sealing ability. Our findings corroborate an earlier study that reported Endosequence bioceramic sealers had superior sealing abilities compared to AH Plus and Epiphany sealers [22]. This is likely due to the hydrophilic properties and expansion during the setting of bioceramic sealers, creating a "self-seal" with up to 0.2% expansion. Additional advantages of bioceramic sealers such as equivalent antibacterial activity, minimal volumetric change, a quick setting time, alkalinization ability, sufficient flow and radiopacity, and less cytotoxicity and genotoxicity as compared to AH Plus sealer have been reported [23,24]. On the contrary, Carvalho et al. found that the biological activity and dentin bond strength were lower for bioceramic sealer as compared to AH Plus [25].

Bioceramic sealer incorporated with chitosan nanoparticles was observed to have the best sealing ability among all the sealers, while canals obturated with gutta percha without sealer exhibited the highest microleakage. This is consistent with the results of the in vitro study by Enggardipta et al., in which the maximum microleakage was observed when obturation was performed with gutta percha without the application of sealer when compared to gutta percha incorporated with epoxy resin or chitosan nanoparticles [1]. The authors concluded that the addition of chitosan particles improved the apical sealing ability of the materials. Furthermore, the antibiotic properties of the sealer are enhanced by the addition of the chitosan nanoparticles, as demonstrated by the complete prevention of the growth of *Enterococcus faecalis* [26,27].

Previous research has also demonstrated that bioceramic sealers excel in dentinal tubule penetration and filling quality, enhancing the apical seal due to its flowability and small particle size [25,28]. They are monomer-free resins that offer advantages such as zero shrinkage, biocompatibility, alkaline pH for hydroxyapatite formation, excellent bonding to gutta percha and dentin, good radiopacity, hydrophilicity, and antibacterial properties [28]. Moisture in dentinal tubules aids its setting reaction, forming hydroxyapatite and a chemical bond with root dentine. Techniques such as photon-induced-photoacoustic streaming and passive ultrasonic irrigation further improve its penetration [29]. However, their removal is challenging in cases of endodontic failure or post-space preparation.

The surface characteristics and hydrophilic nature of the sealers incorporated with nanoparticles significantly influence their wetting ability and adherence to irregular dentin surfaces when incorporated into root canal sealers. Chitosan nanoparticles, being hydrophilic, enhance their absorption and interaction with dentin, improving the sealing ability of bioceramic sealers [30]. The improved adhesiveness occurs as a result of electrostatic bonding, in which the chitosan amine group (NH3 +) is attracted by the collagen carboxyl group (COO−) [31] This enhanced interaction of the amino and hydroxyl groups of the chitosan molecule promotes cationic properties and ionic interactions with dentin calcium ions, facilitating deeper penetration into dentinal tubules. Moreover, the nanosized particles of chitosan increase the surface area-to-volume ratio, further enhancing their hydrophilic characteristics and overall sealing effectiveness. These findings parallel those of Yehia et al., who demonstrated that nanoparticles can enhance the antimicrobial efficacy and adaptability of bioceramic sealers to root canal dentin, highlighting their potential in improving sealer performance [27].

The results of this study underscore the clinical implications of using bioceramic sealers, particularly those incorporated with chitosan nanoparticles, in reducing microleakage and enhancing the apical seal. These improvements are critical in minimizing pathways for bacterial ingress, a primary factor in endodontic re-infections. The superior sealing ability of bioceramic-chitosan nanoparticle sealers could significantly contribute to the long-term success of root canal treatments by limiting residual microbial activity and preventing reinfection of the periapical tissues. Additionally, the enhanced antibacterial properties of chitosan nanoparticles, particularly against persistent pathogens such as *Enterococcus faecalis*, provide an added advantage in achieving a sterile root canal environment. These benefits suggest that incorporating such advanced materials into clinical practice could reduce retreatment rates, improve patient outcomes, and enhance overall treatment predictability in endodontics.

Certain limitations of the present study must be considered when generalizing the results to real clinical settings. Since the study design was in vitro, the microleakage occurring in the samples may or may not represent the true extent occurring in the oral cavity given the fact that there are several factors influencing microleakage and fluid dynamics in the oral cavity. The extracted tooth samples and in vitro settings may not be able to precisely simulate the oral environment. Even so, findings of the present study support that incorporating chitosan nanoparticles in bioceramic sealer enhances its properties. The material can be used in root canal obturation with good sealing ability so that apical leakage can be minimized, resulting in enhanced treatment outcomes.

The findings of this study highlight the potential clinical advantages of using bioceramic sealers, especially those enhanced with chitosan nanoparticles, in achieving superior apical sealing and reducing microleakage. These attributes could translate to improved long-term clinical outcomes such as reduced risk of reinfection and enhanced success rates of root canal therapy. However, the long-term effects of these materials on the surrounding periapical tissues and their biocompatibility in dynamic oral environments need further exploration. While bioceramic sealers are known for their biocompatibility, additional clinical trials are necessary to assess their performance over extended periods, particularly in cases of retreatment or post-space preparation. Furthermore, factors such as the influence of masticatory forces, thermal cycling, and the interaction of sealers with irrigants and medicaments in vivo should be considered. Future research should focus on investigating these aspects through long-term clinical studies and biocompatibility assessments to validate the utility of these materials in routine endodontic practice.

## Conclusions

The findings of this study demonstrate that the incorporation of chitosan nanoparticles into bioceramic sealers significantly enhances their sealing ability, resulting in reduced apical microleakage compared to both AH Plus sealer and bioceramic sealer alone. The superior performance of the bioceramic sealer with chitosan nanoparticles highlights its potential as an effective material for achieving a more reliable seal in root canal obturation, thereby minimizing the risk of endodontic re-infections. Given the promising results, the use of chitosan nanoparticles in endodontic sealers offers a valuable advancement in improving the quality of endodontic treatments and enhancing patient outcomes. Future research should explore the long-term clinical performance and biocompatibility of this novel sealer to further validate its application in endodontics.

## Figures and Tables

**Figure 4:**
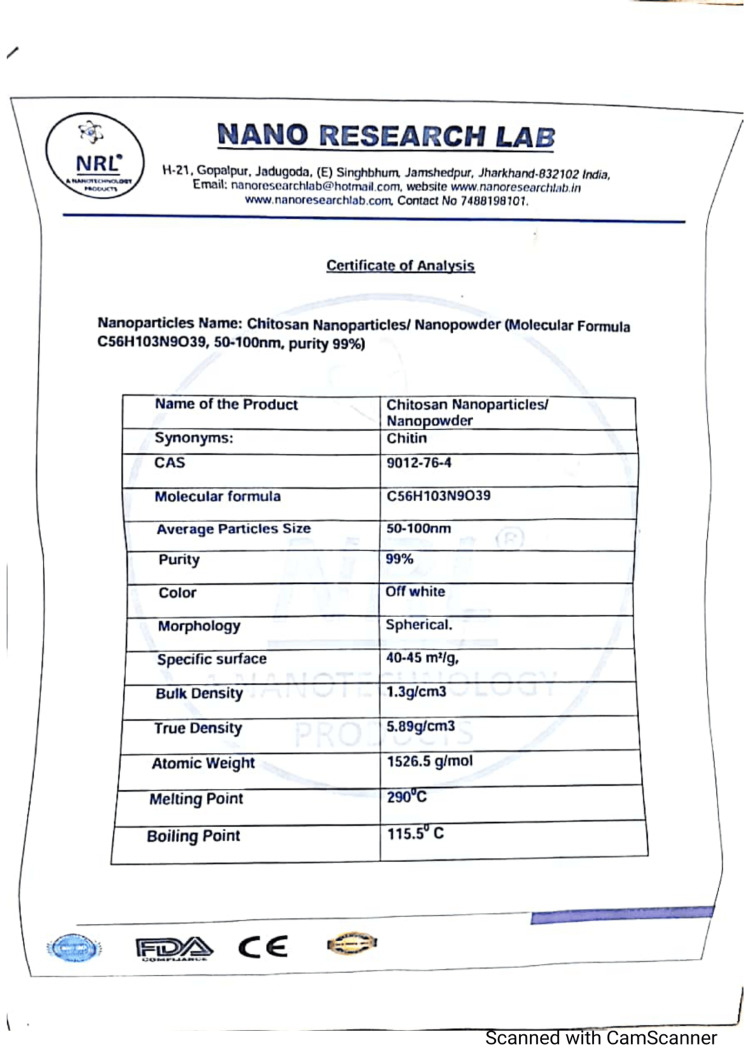
Certificate of analysis of the characteristics of chitosan particles
